# Comparison of Preoperative Cone-Beam Computed Tomography and 3D-Double Echo Steady-State MRI in Third Molar Surgery

**DOI:** 10.3390/jcm10204768

**Published:** 2021-10-18

**Authors:** Silvio Valdec, Adib Al-Haj Husain, Sebastian Winklhofer, Marcel Müller, Marco Piccirelli, Bernd Stadlinger

**Affiliations:** 1 Clinic of Cranio-Maxillofacial and Oral Surgery, Center of Dental Medicine, University of Zurich, 8032 Zurich, Switzerland; adib.alhaj@gmail.com (A.A.-H.H.); bernd.stadlinger@zzm.uzh.ch (B.S.); 2 Department of Stomatology, Division of Periodontology, Dental School, University of São Paulo, Butantã 2227, SP, Brazil; 3 Department of Neuroradiology, Clinical Neuroscience Center, University Hospital of Zurich, University of Zurich, 8091 Zurich, Switzerland; sebastian.winklhofer@usz.ch (S.W.); marco.piccirelli@usz.ch (M.P.); 4 Statistical Services, Center of Dental Medicine, University of Zurich, 8032 Zurich, Switzerland; marcel.mueller@zzm.uzh.ch

**Keywords:** cone-beam computed tomography, inferior alveolar nerve, magnetic resonance imaging, oral surgery, oral anatomy

## Abstract

We investigated the reliability of assessing a positional relationship between the inferior alveolar nerve (IAN) and mandibular third molar (MTM) based on CBCT, 3D-DESS MRI, and CBCT/MRI image fusion. Furthermore, we evaluated qualitative parameters such as inflammatory processes and imaging fusion patterns. Therefore, two raters prospectively assessed in 19 patients with high-risk MTM surgery cases several parameters for technical image quality and diagnostic ability using modified Likert rating scales. Inter- and intra-reader agreement was evaluated by performing weighted kappa analysis. The inter- and intra-reader agreement for the positional relationship was moderate (κ = 0.566, κ = 0.577). Regarding the detectability of inflammatory processes, the agreement was substantial (κ = 0.66, κ = 0.668), with MRI providing a superior diagnostic benefit regarding early inflammation detection. Independent of the readers’ experience, the agreement of judgment in 3D-DESS MRI was adequate. Black bone MRI sequences such as 3D-DESS MRI providing highly confidential preoperative assessment in MTM surgery have no significant limitations in diagnostic information. With improved cost and time efficiency, dental MRI has the potential to establish itself as a valid alternative in high-risk cases compared to CBCT in future clinical routine.

## 1. Introduction

The prevention of inferior alveolar nerve (IAN) damage when performing mandibular third molar (MTM) surgery near the mandibular canal (MC) in high-risk cases is of fundamental importance in everyday clinical practice. Therefore, selecting an accurate, reliable imaging modality prior to MTM surgery is essential to avoid unpleasant postoperative complications. The incidence of iatrogenic injuries to the IAN in the surgical removal of MTM is estimated in the literature to be around 4% (0.4–8.4%) [1,2], whereby radiographic signs, such as a close positional relationship between the MTM and the MC [3] and incomplete integrity of the MC’s osseous boundaries [4] increase the probability of IAN damage. Permanent neurosensory disturbances are often accompanied by psychological and social impairments, which significantly decrease the quality of life of affected patients [5].

Hence, before the surgical intervention, the widely used conventional two-dimensional panoramic radiography (PAN) is routinely assessed, providing anatomical information about angulation and position of the MTM and the spatial proximity between the roots of the MTM and the MC. Two-dimensional imaging is not sufficient in cases where radiographic features such as the superimposition of anatomical structures, darkening of the MTM’s roots, non-continuous cortical integrity, or diversion of the MC are displayed [6]. For this purpose, cone-beam computed tomography (CBCT) is indicated, providing three-dimensional information about the accurate buccolingual localization of the MC, visualization of its osseous cortical boundaries, and the number and shape of the MTM’s roots [7,8]. CBCT is considered the gold standard in three-dimensional dentomaxillofacial imaging for hard tissues with advantages such as lower radiation exposure and lower costs compared to conventional computed tomography (CT) [9]. Advances in CBCT diagnostics have further improved MTM surgery in general toward a more thorough planning removal strategy. Additionally, they have also provided alternative treatment options such as coronectomy to exodontia [10]. Nevertheless, the use of CBCT entails certain disadvantages, such as deficiencies in soft-tissue contrast, uniform grayscale values [9], and high radiation exposure especially for the thyroid [11]. Considering the increased use of CBCT in dental treatments of younger, radiosensitive patients, there might be an association with an increased possibility of radiation-induced cancer [11,12]. Since the risk of radiation-induced cancer is cumulative over a lifetime [11,13], radiation minimization or even elimination should be aimed in future dental clinical routines.

As conventional X-ray-based imaging modalities such as PAN and CBCT can only visualize the MC’s osseous boundaries, magnetic resonance imaging (MRI) with its excellent soft-tissue contrast is capable of providing direct visualization of the IAN [14]. Despite some limitations concerning hard tissue contrast and susceptibility to motion artifacts, MRI has become a promising imaging modality in the head and neck region in an increasing number of indications. The remaining challenges in oral cavity imaging by MRI are field inhomogeneity, dental implants, or artifact inducing, metal-containing dental restorations [15,16].

A growing number of clinical MRI studies have investigated the IAN’s visualization in general and its positional relationship to the MTM, with reports achieving promising results in the context of preoperative imaging assessment in MTM surgery [17,18]. Recently applied “black bone” sequences such as 3D-Double Echo Steady State (DESS) and 3D Short-TI Inversion Recovery (STIR) assessed good feasibility and excellent visualization of the IAN [15,19,20,21]. In this specific MRI protocol, the neural tissue of the IAN appears as a high signal intensity structure and could be distinguished from the mandibular canal due to the myelin layer or the bone marrow surrounding the nerve, allowing excellent visualization by application of the water excitation fat-suppression technique [22].

This study aimed to investigate the reliability in assessing the positional relationship between the IAN, respectively MC and the roots of complex MTMs and the assessment of qualitative parameters such as inflammatory processes and fusion patterns based on CBCT, 3D-DESS MRI, and CBCT/MRI image fusion.

## 2. Materials and Methods

### 2.1. Study Design

This prospective clinical cohort study was designed and realized in collaboration between the Clinic of Cranio-Maxillofacial and Oral Surgery of the Center of Dental Medicine (University of Zurich) and the Department of Neuroradiology of the University Hospital of Zurich (University of Zurich). The main goal of this study was to assess qualitative parameters, such as the positional relationship between the IAN/MC and the MTMs, the presence of inflammatory processes, and fusion patterns based on different imaging modalities. The study’s inclusion criteria were defined as follows: (1) age between 18 and 65 years; (2) indication for extraction of partially retained, fully retained, or impacted mandibular third molars; and (3) positional relationship between the MTM and the MC/IAN indicating three-dimensional imaging. The exclusion criteria were (4) acute odontogenic infection; (5) nerve damage to the three large branches of the trigeminal nerve (ophthalmic branch (V1), maxillary branch (V2), and mandibular branch (V3); (6) adjacent implants or metallic reconstructions; and (7) common contraindication for MRI imaging such as pregnancy, claustrophobia, anxiety, metallic intraocular foreign bodies, cerebral aneurysm clips in the brain, and cardiovascular implantable electronic devices.

**Figure 1:**
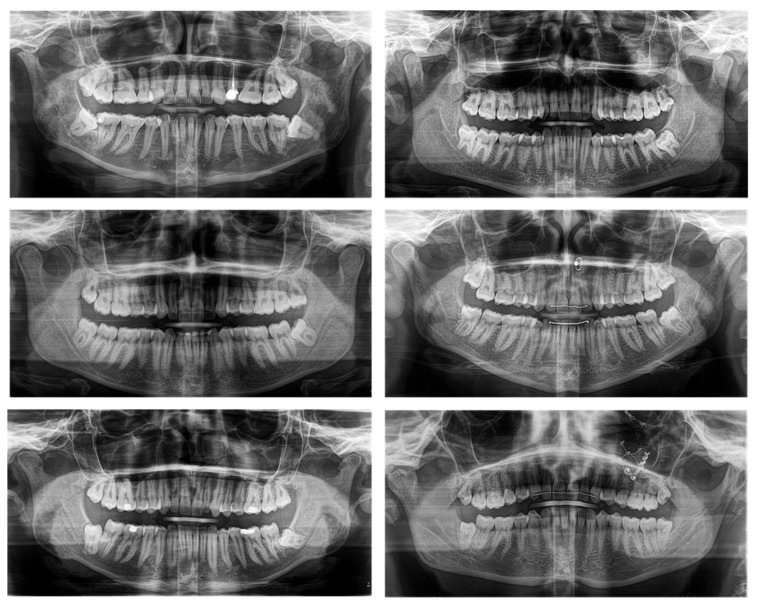
Study participants’ two-dimensional panoramic radiography (PAN) providing anatomical information about the angulation and position of the mandibular third molars (MTM) and the spatial proximity between the roots of the MTMs and the mandibular canal (MC). All participants showed an indication for the removal of MTMs and a close relationship between the MC and the MTM; therefore, these were considered high-risk cases and show indication for three-dimensional imaging.

The estimated number to be enrolled in 8 months was 20, whereby the study participants’ recruitment took place between May 2018 and December 2018, with an indication for the removal of MTMs, showing a close positional relationship between the IAN/MC and the roots of the MTMs in PAN indicating three-dimensional imaging according to the guidelines of the Swiss association of dentomaxillofacial radiology and therefore considered as high-risk cases [6] (Figure 1).

The referral to the Clinic of Cranio-Maxillofacial and Oral Surgery of the Center of Dental Medicine (University of Zurich) was made by their general dental practitioner or self-referral. Before surgical extraction, all study participants underwent CBCT and MRI scans. Trained neuroradiologists obtained MRI acquisition, and CBCT image acquisition was performed by trained research personnel of the Clinic of Cranio-Maxillofacial and Oral Surgery. Experienced oral surgeons performed subsequent MTM surgery.

Reporting complies to the STROBE (“Strengthening the Reporting of Observational studies in Epidemiology”) guidelines.

### 2.2. MRI Data Acquisition

All study participants underwent MRI examination prior to surgical MTM removal on a 3 Tesla Skyra (release VE11c, Siemens Healthineers, Erlangen, Germany) using a Siemens standard 64 channel head-and-neck coil. The used axial 3D-DESS with water excitation MRI sequence had an isotropic acquisition resolution of 0.75 × 0.75 × 0.75 mm^3^ together with a receive bandwidth of 355 Hz/Px. The other sequence specifications were field-of-view 242 × 242 × 78 mm^3^; acquisition matrix 320 × 320 × 104; slice oversampling 100%; no parallel acquisition; one signal average; acquisition time 12:24 min:s; TR/TE1/TE2 11.2/4.2/7.7 ms; flip angle 30°; selective water excitation.

### 2.3. CBCT Data Acquisition

The Orthophos SL 3D scanner (Dentsply Sirona, Bensheim, Germany) was applied for CBCT data collection. The ideal reproducible head positioning of each study participant was achieved using the positioning laser beams of the scanner. Simultaneously, adjusted head supports and chin rests were used during CBCT scanning time. The CBCT routine clinical imaging protocol defined by the following specifications was applied: 85 kV; 13 mA; radiation time 4.4 s; voxel size 160 μm; FOV 11 × 10 cm.

### 2.4. Image Evaluation

Storage and analysis of the CBCT and 3D-DESS MRI DICOM data were performed in the local Picture Archiving and Communication System (PACS) (IMPAX EE R20, release XV, Agfa Healthcare, Mortsel, Belgium) and OnDemand3D (Cybermed, Seoul, Korea). Two readers (reader A, attending board certified oral surgeon, and reader B, fifth-year dental medicine student) with varying degrees of experience performed image analysis on axial, coronal, and sagittal DESS multiplanar image reconstructions (MPR) and maximum intensity projections (MIP) on a 2-megapixel high-resolution liquid-crystal display. Before performing the evaluation, both readers conducted a calibration session in which some randomly selected cases were assessed to resolve any ambiguity. All readers were allowed to use all DICOM Viewer functions to adjust image parameters such as magnification, contrast, and brightness to optimize the image display. All readers were blinded to each other’s results and earlier performed their own readouts. Inter- and intra-rater agreements were assessed. Recall bias was avoided by having the readers repeat the readout in a random order after a time interval of 4 weeks.

Technical image quality and diagnostic capability were evaluated in terms of diagnostic confidence, background noise, and image resolution using a modified 5-point Likert rating scale for the DESS sequence according to Burian et al. [20]: 1, very poor, not suitable for clinical use; 2, poor, substantial adverse effect for clinical use; 3, average, borderline clinical use due to the image quality; 4, very good, containing no substantial adverse effect for clinical use; 5, excellent, no restrictions for clinical use. A 4-point Likert rating scale assessed artifacts in the mandibular molar region: 0, massive artifacts caused by dental restorations (high); 1, moderate artifacts caused by dental restorations (moderate); 2, minor artifacts caused by dental restorations (low); 3, absence of artifacts caused by dental restorations (none). Additionally, the continuous detectability of the delimitation of the MC’s osseous cortical boundaries in the CBCT and the high signal intensity structure of the IAN in MRI were assessed for the retromolar and molar regions. According to the evaluation method of Deepho et al. [23], the MC was considered as visible if its osseous cortical boundaries were seen as a circle on the cross-sectional coronal reconstruction and as invisible if a part or even the complete osseous cortical boundaries was missing. The same evaluation scale was also applied for the DESS images with the IAN to be considered visible if the IAN’s high signal intensity course could be followed continuously and as invisible if an interruption in the course of the IAN could be detected.

**Figure 2:**
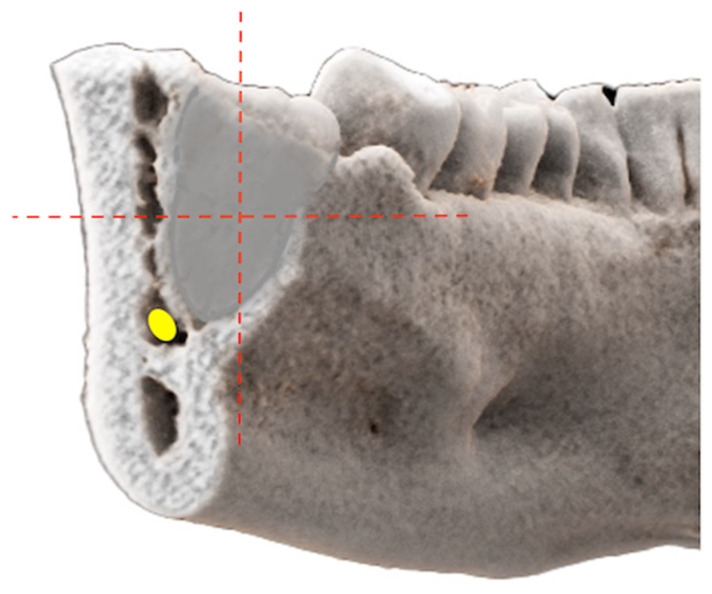
Photorealistic three-dimensional (3D) visualization of a study participant’s cone-beam computed tomography (CBCT) using cinematic rendering (CR) image reconstructions. In the coronal reference layer, showing the closest positional relationship, both readers independently classified the relative positional relationship of the mandibular third molar’s (MTM) roots and the mandibular canal (MC)/inferior alveolar nerve (IAN) by using the Cartesian coordinate system, according to Wang et al. [24] After evaluating whether there was contact or not, the structural center of the MTM was set as the origin in the Cartesian coordinate system and the relative positional relationship—whether the IAN’s position was periradicular (lingual, buccal, inferior) or interradicular (between the roots)—was determined.

In the coronal reference layer, showing the closest positional relationship, both readers independently classified the relative positional relationship of the MTM’s roots and the MC/IAN by using the cartesian coordinate system, according to Wang et al. [24] (Figure 2).

**Figure 3:**
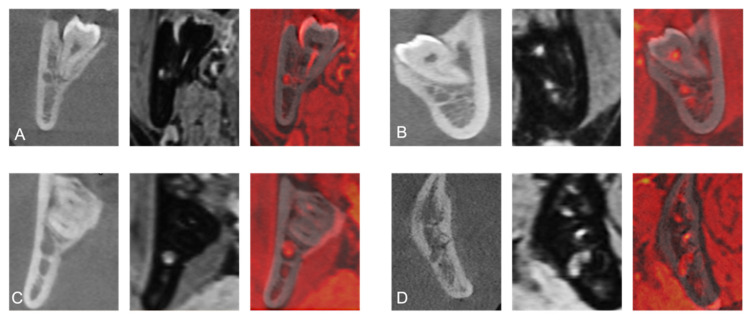
Coronal reconstruction of a study participant’s CBCT, 3D-DESS MRI, and fused CBCT/MRI images visualizing periradicular (buccal (**A**); lingual (**B**); inferior (**C**)) or interradicular (between the roots (**D**)) relative positional relationship of the IAN at the third molar.

After evaluating whether there was a contact or not, the structural center of the MTM was set as the center of the Cartesian coordinate system. The exact relative positional relationship was determined whether the IAN’s position was periradicular (lingual, buccal, inferior) or interradicular (between the roots) (Figure 3).

In addition, all cases were examined by MRI for the presence of inflammatory processes of the soft tissues and their imaging mimics, using the following qualitative classification: yes, probably yes, and no.

**Figure 4:**
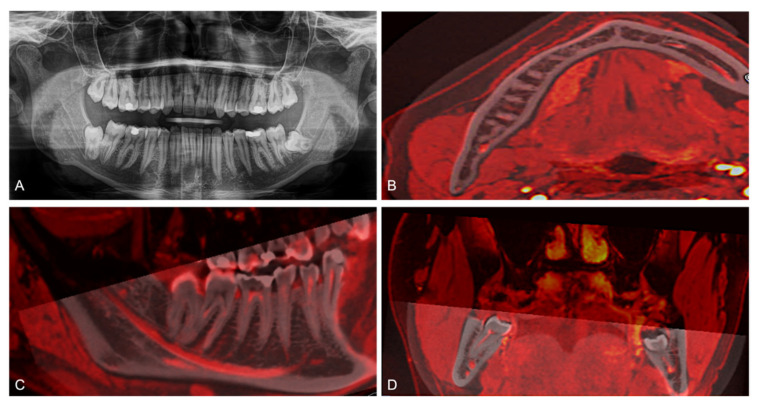
(**A**) Two-dimensional panoramic radiography (PAN) of a study participant, CBCT and MRI scans of a study participant were transferred to OnDemand3D and fused using the “automatic registration” function at an equal slice thickness of 0.5 mm. The MRI scan was labeled red, and conventional grayscale values displayed the CBCT scan. Axial (**B**), sagittal (**C**), and coronal (**D**) fused CBCT/MRI images are visualized.

CBCT and MRI scans of each study participant were transferred to OnDemand3D and fused using the “automatic registration” function at an equal slice thickness of 0.5 mm. The MRI scan was labeled red, and conventional grayscale values displayed the CBCT scan. If the fusion was rated as not perfect, in all cases, these images were manually re-registered in consensus using trial and error until a complete overlapping in the coronal, axial, and sagittal planes was achieved. The final CBCT/3D-DESS MRI volumetric fusions were used for evaluation by defining the retromolar and molar region as regions of interest (Figure 4).

**Figure 5:**
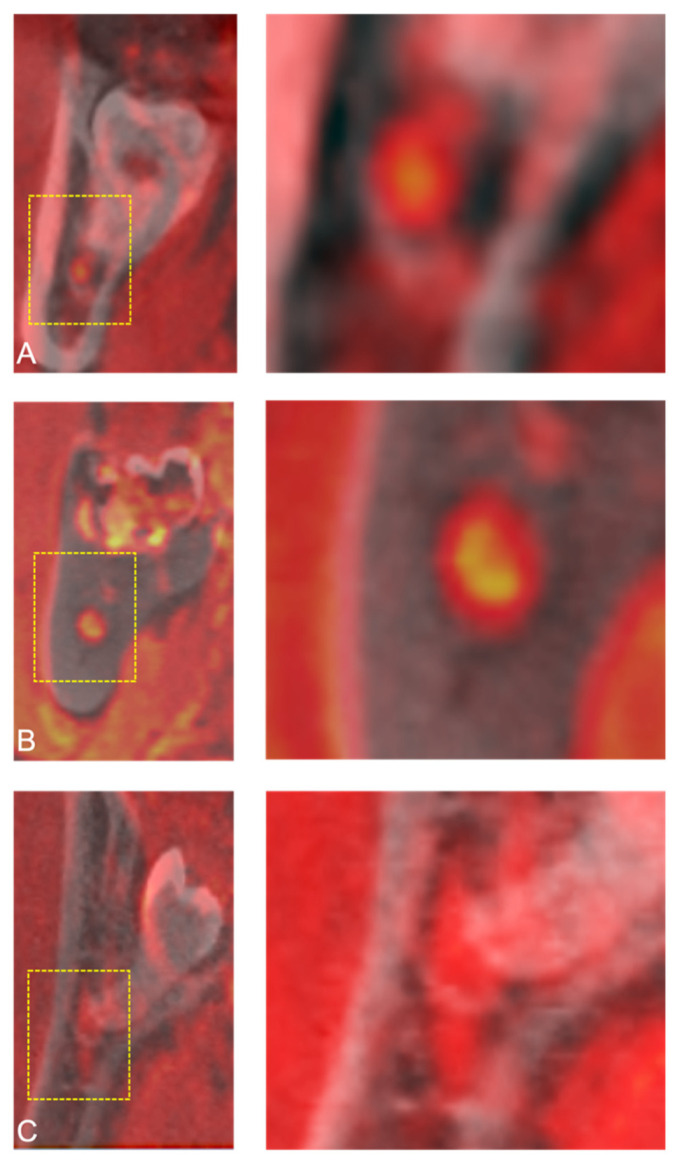
To investigate the occupation of the mandibular canal (MC) by the inferior alveolar nerve’s (IAN) hyperintense signals, the following three patterns were determined according to Deepho et al. [23]: (**A**) small pattern, if the area within the MC was only partially occupied by the presence of IAN MRI signal hyperintensities; (**B**) same pattern, if the area within the MC was occupied by the presence of IAN MRI signal hyperintensities; and (**C**) large pattern, if the IAN was sticking out of the MC area.

To investigate whether there are MRI related distortions and how the occupation of the MC by the IAN’s hyperintense signals varies over the defined regions of interest, the following three patterns were determined by both readers according to Deepho et al. [23]: same pattern, if the area within the MC was occupied by the presence of IAN MRI signal hyperintensities; small pattern, if the area within the MC was only partially occupied by the presence of IAN MRI signal hyperintensities; and large pattern, if the IAN was sticking out of the MC area (Figure 5).

### 2.5. Statistical Analysis

The statistical analysis of the acquired data was performed using the statistical software R 4.0.5, including the packages irr, vcd, and ggplot2 (R Core Team. R: A Language and Environment for Statistical Computing; R Foundation for Statistical Computing: Vienna, Austria). The mean and standard deviation was calculated over reader, readouts, sides, and subjects for the technical image quality, artifacts, and the MC and IAN visibility. The reliability of the positional relationship between the MC/IAN and the MTM and the presence of inflammatory processes were assessed by bootstrap resampling. Kappa coefficients and corresponding 95% confidence intervals (CI) were reported for the two readouts and two readers via a separate inter-rater and intra-rater reliability coefficient. A resampling approach was performed, whereby the calculation of the kappa coefficients was repeated 1000 times. Each time, a new dataset was created from the original data by randomly using one value for each combination of subject, reader, and readout from the values observed for the left and right side. Then, the reported value for inter- and intra-rater reliability and the associated 95% CIs were obtained from taking the mean over the 1000 corresponding calculated statistics. Intra- and interobserver variability of the fusion pattern analysis was assessed using squared weighted kappa analysis. The strength of agreement beyond chance for the obtained weighted kappa coefficients can be interpreted as follows: poor, <0; slight, 0–0.2; fair, 0.21–0.4; moderate, 0.41–0.6; substantial, 0.61–0.8; almost perfect 0.81–1 [25]. To investigate whether both imaging modalities showed significant differences, a one-sample Wilcoxon signed-rank test was performed by testing the null hypothesis “the difference in the expected realization between both imaging modalities is zero”. We reject the null hypothesis based on a specified significance level of 5%. Conversely, when rejecting the null hypothesis, this could indicate that there is a significant difference between the two imaging modalities with respect to the tested variable.

Post hoc sample size calculation was performed for the categorical variable positional relationship, whereby the present dataset was used to obtain the prevalence of various possible positional relationships, which might be relevant for future research projects. The sample size calculation refers to the interrater reliability for MRI, whereby disagreements are weighted according to their simple distance to perfect agreement due to dealing with a nominal variable [26]. Thereby, the kappa value for an unacceptable agreement was set to 0.4 and that for an acceptable agreement was set to 0.6. The significance level was set at 5% and the desired power was set at 80%. The relative frequency estimates of the occurrence of each categorical level were calculated based on the same resampling approach described above on the current dataset. The aggregated required post hoc sample size corresponds to the median of all 1000 calculated sample sizes and is accompanied by the associated empirical 95% CI.

## 3. Results

Twenty-three patients (six male, 13 female) with a mean age of 30.5 ± 13 years (median age, 25 years; age range, 18–63 years) were enrolled for 8 months. Four participants did not show up for the CBCT or MRI scan appointment; therefore, 19 participants were evaluated (this results in 36 inferior alveolar nerves, in two cases, the mandibular third molar was missing). Technical image quality was very good in CBCT (4.86 ± 0.37, inter-reader κ = 0.872 (95% CI: 0.7–1), intra-reader κ = 0.812 (95% CI: 0.63–0.99)) and MRI (4.72 ± 0.53, inter-reader κ = 0.945 (95% CI: 0.91–0.98), intra-reader κ = 0.877 (95% CI: 0.72–0.98)), indicating no substantial adverse effect for clinical use. In general, no artifacts in the retromolar and molar region provoked by dental restorations were noted with a mean value of 2.92 ± 0.28 (inter-reader κ = 0.818 (95% CI: 0.75–0.89), intra-reader κ = 0.742 (95% CI: 0.6–0.89)) in CBCT and 2.75 ± 0.47 (inter-reader κ = 0.911 (95% CI: 0.81–0.99), intra-reader κ = 0.804 (95% CI: 0.58–1)) in MRI. Both imaging modalities showed no significant differences regarding imaging quality (*p* = 0.342) and artifacts (*p* = 0.057). In all cases, the MRI high signal intensity structures of the IAN and its surrounding osseous boundaries were consistently visible; however, in 7.2% of the cases a non-continuous detectability of the MC was observed in the retromolar and molar CBCT images.

**Figure 6:**
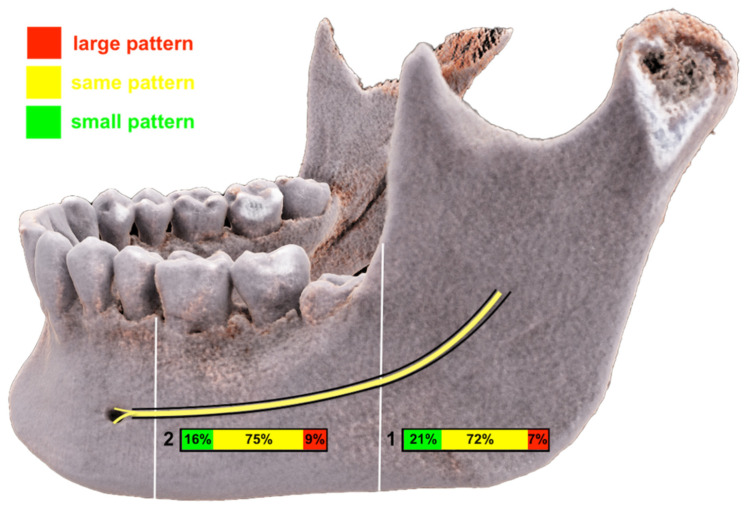
Photorealistic three-dimensional (3D) visualization of a study participant’s cone beam computed tomography (CBCT) using cinematic rendering (CR) image reconstructions, visualizing the quantitative evaluation of the imaging fusion pattern in the retromolar and molar region.

Regarding the classification of the positional relationship between the IAN/MC and the MTM, the mean agreement was moderate in CBCT (inter-reader κ = 0.566, intra-reader κ = 0.577) and MRI (inter-reader κ = 0.568, intra-reader κ = 0.524) (Figure 6, Table 1).

The presence of inflammatory processes showed a substantial mean agreement (inter-reader κ = 0.66, intra-reader κ = 0.668) (Table 2).

The additional examination of the inter- and intra-reader agreement regarding the fusion pattern analysis was almost perfect for the retromolar region (inter-reader κ = 0.975, intra-reader κ = 0.828) and moderate in the molar region (inter-reader κ = 0.526, intra-reader κ = 0.476) (Table 3).

## 4. Discussion

The selection of the most appropriate three-dimensional imaging modality before MTM removal in high-risk cases associated with a risk for IAN damage remains a challenge in everyday clinical practice. Therefore, this prospective cohort study aimed to evaluate 3D-DESS MRI as a possible alternative or supplement to CBCT in decision making and planning prior to MTM surgery in complex cases.

**Figure 7:**
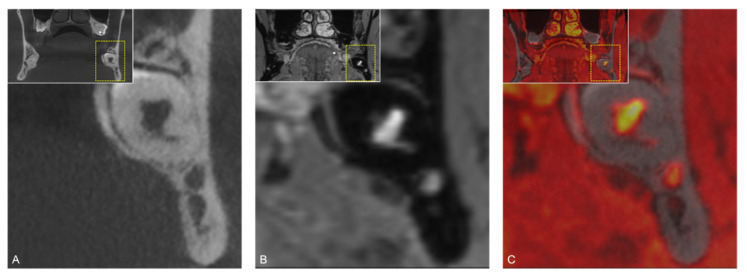
(**A**) CBCT, (**B**) MRI, and (**C**) fused CBCT/MRI scans of the positional relationship between the mandibular canal (MC)/inferior alveolar nerve (IAN) and the mandibular third molar (MTM). This borderline case visualizes the advantages of the selected imaging modality and their respective limitations. Additional relevant information, whether direct visualization of the IAN is possible or only the bony borders of the MC can be displayed, is presented depending on the imaging modality.

This study demonstrated the feasibility of accurately displaying the close relationship between the IAN/MC and MTM in both imaging modalities with excellent image quality and typically in the absence of artifacts. Regarding the positional relationship, a moderate inter- and intra-reader agreement in CBCT and MRI could be observed regardless of the examiners’ experience. To the authors’ knowledge, only a few studies have compared the imaging modalities CBCT and MRI in the context of IAN and MC visualization, with only one explicitly assessing the positional relationship between the MTM and IAN using standard MRI protocols of the jaw region [27]. The results obtained showed that MRI might be a variable alternative in dental imaging. The observed differences in this study between the different imaging modalities (in 8.5% of the cases) could be found mainly in the contact/non-contact cases, confirming the findings of Beck et al. in their previously conducted comparison study [27]. This may be related to the selected imaging modality and its limitations, whether direct visualization of the IAN is possible or only the osseous boundaries of the MC can be visualized (Figure 7).

This aspect was mainly registered in borderline cases where no osseous boundary of the MC is displayed in CBCT, but where the MR imaging still demonstrates a distance between the MTM’s roots and the IAN. In addition, a disagreement of judgment was noted in cases where buccal/lingual and inferior position classification was interpreted differently. In this study, a slightly lower inter- and intra-reader agreement was observed compared to previous reports [27]. Generally, the disagreements observed can be explained by the sample size of the study population, which included almost exclusively borderline cases, by the inexperience of MRI interpretation by dental clinicians, or by the statistical evaluation method that used bootstrap resampling. Previously conducted studies proved that specific radiographic signs, such as interruption of the MC and darkening, deflection, diversion, or narrowing of the MTM roots, are associated with a significantly increased risk for IAN damage in MTM surgery [28]. Additional factors potentially having a significant impact on the postoperative outcome are age, gender, development of the MTM’s roots, degree of impaction, and medical history [29,30]. Three-dimensional X-ray-based imaging modalities can only display the osseous cortical boundaries of the MC, depending on its trabecular quality and density [31]. The MC cannot be perfectly visualized in CBCT in up to 25% of the cases, whereby the distinction of the MC from the adjacent tissues becomes more apparent in the distal segment of the mandible [32]. Other reports proclaim difficulties in displaying the MC in the first molar region in cases with a clear-cut bony delimitation of the MC using CBCT [33]. The results of this study confirmed these findings, as the visibility of the MC was given in 94.8% of the cases in the retromolar and in 90.8% in the molar region with excellent image quality, showing no restrictions for clinical use.

Considering the frequency of MTM surgery, the additional annual increase in radiation-induced cancer incidence by 0.46 due to CBCT application before MTM surgery [34], and the controversial benefit of CBCT imaging in comparison to conventional PAN in terms of decreasing postoperative neurosensory impairments [35,36], MRI is of interest as a valid alternative. However, using high-resolution MRI, the IAN can be directly visualized without radiation exposure, irrespective of the integrity of the MC’s cortical boundaries [37]. An increasing number of MRI studies directly depicted the IAN, with some performed reports achieving promising results in the context of preoperative radiological assessment in MTM surgery [17,18]. The results obtained by 3 Tesla MRI provided much better visualization of the cranial nerves than 1.5 Tesla MRI, whereas simultaneously an accurate visualization of the IAN was observed [38]. Recently applied “black bone” MRI protocols such as 3D-DESS and 3D STIR demonstrated excellent visualization of the peripheral branches of the mandibular nerve, specifically for the IAN and lingual nerve (LN) [15,19,20,21,39]. The 3D-DESS MRI sequence, currently one of the routine clinical MRI protocols in musculoskeletal imaging, localization of the facial nerve, and parotid tumors, allows accurately displaying the IAN due to the lipid-rich myelin layer surrounding it [22]. This study confirmed previous findings of accurately visualizing the IAN’s tissue within the osseous boundaries of the MC [15,19,20,40]. More precisely, high-resolution MRI displays the entire neurovascular bundle (NVB) composed of the IAN, which is divided into a larger mental nerve and a smaller incisive nerve [41], and the inferior alveolar artery (IAA), which is located within the MC [42]. Yu et al. showed in a histomorphometric analysis of the NVB at the MTM level that the IAN represented 32.4% and the IAA represented 4.5% of the area [43]. Hence, it can be assumed that the seven times larger IAN is mainly visualized on MRI. In addition, several reports state that the MC in the MTM region has even higher detectability in MRI compared to CT [33] and CBCT [44]. This study’s results support these statements, as in all cases, the IAN and the MC could be visualized. Considering all these aspects, this could explain the disagreement of assessing the positional relationship in borderline cases where no osseous boundary of the MC is displayed in CBCT, but the MRI imaging still demonstrates a distance between the MTM’s roots and the IAN.

**Figure 8:**
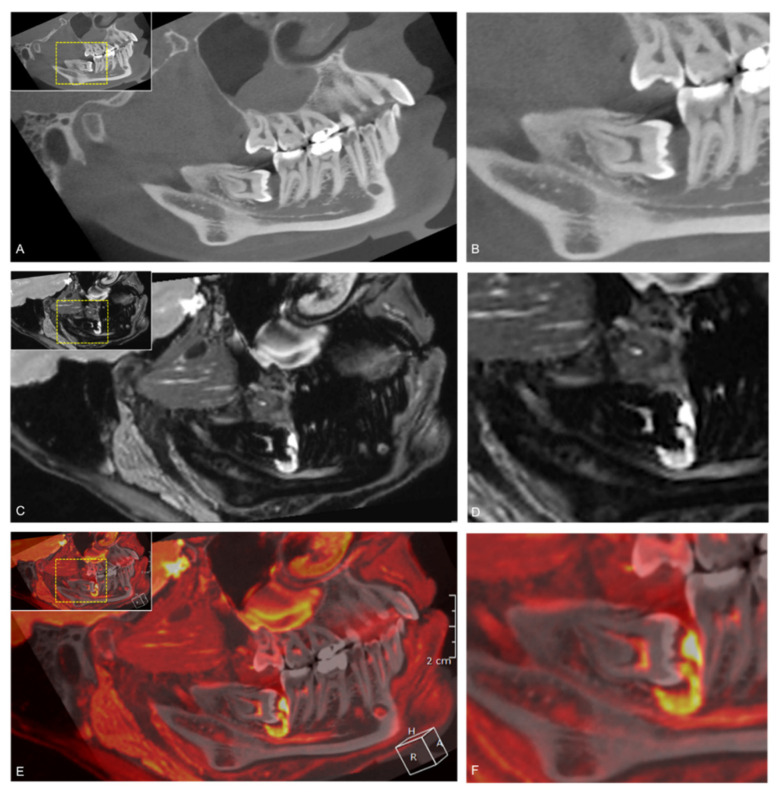
Fusion of study participant’s 3D-DESS MRI (red) and CBCT (gray). (**A**) Sagittal CBCT reconstruction showing an impacted mandibular third molar with mesial angulation and surrounding follicular cyst; (**C**) Sagittal DESS MRI visualizing an inflammatory process originating from the follicular cyst, which impressively displaces the IAN within the IAC; and (**E**) Fused CBCT-MRI scans visualizing the inflammatory process. For orientation, the dotted rectangles in the corner show the enlarged area in CBCT (**B**), MRI (**D**), and fused CBCT/MRI scans (**F**).

Taking into account the superior visualization of bony tissue in CT (respectively CBCT) in the dentomaxillofacial field and that the NVB does not always completely fill the MC, CBCT and MRI scans were fused to determine the fusion pattern in the retromolar and molar region and to investigate whether an additional diagnostic benefit could be achieved. This study confirms the trends observed by Deepho et al. that there is a tendency for the “small pattern” (21%) to be more prevalent in the retromolar region. Nevertheless, it should be noted that the “same pattern” was most frequent in the retromolar (72%) and molar (76%) regions [23]. The large pattern was observed in only 7% of the cases, which is most likely explained by insufficient fusion or insufficient MRI image quality due to motion artifacts. The IAN not filling the MC’s complete volume could explain the different positional relationships in both image modalities due to the minimal difference in IAN position in MRI images compared to CBCT images. It is essential to consider that the fused CBCT/MRI images in this study could not be obtained only by automatic registration function. It was necessary to correct the fusion manually through trial and error to allow the best possible evaluation. Therefore, we can only confirm the need for a perfect automatic fusion algorithm from other studies [23]. However, compared to the MRI protocols used in other studies, we achieved a much better image quality and more accurate evaluation using 3D-DESS MRI protocol. Compared to CT or MRI imaging alone, the use of fused CBCT/MRI images in these borderline cases might offer advantages to the standard preoperative radiological assessment. In cases where inflammatory processes could be detected in the MTM region, MRI and fused CBCT/MRI scans provide a diagnostic advantage with substantial reliability (inter-reader κ = 0.66, intra-reader κ = 0.668), independent of the readers’ experience. This confirms the trends of previous studies that MR imaging is superior in visualizing soft tissue disease, such as periodontitis, even before any osseous defect occurred (Figure 8) [45].

There is only one study comparing the positional relationship in MRI and CBCT imaging in the context of MTM surgery using a standard radiology protocol (axial PD T2 TSE and coronal PD TSE) of the jaw region. However, the 3D-DESS MRI protocol, a specific sequence enabling good feasibility and excellent visualization of the IAN, applied in this study, could confirm the trends of Beck et al. regarding reliability in preoperative radiological assessment [27]. The difficulties found in determining the positional relationship of complex borderline cases remained despite using the DESS MRI sequence. However, high-resolution and high-contrast images allowing simultaneous visualization of the IAN’s nerve tissue within the MC’s osseous boundaries could be obtained, providing an advantage in determining whether there is a contact or non-contact case. As this MRI protocol can also be applied to various other surgical procedures with higher complexity in the oral cavity, such as maxillary sinus elevation or the guided placement of dental implants, and can be combined with other digital images, such as intraoral scans [46], further studies should evaluate the correlation between the chosen preoperative imaging modality and surgical management and the associated potential IAN damage.

There are several limitations in this study. First, there is a limitation due to the small sample size. Hence, it is challenging to provide generally valid statements. Further studies with larger cohorts are needed to confirm the trends obtained by using the 3D-DESS MRI protocol in preoperative radiological assessment with high reliability and validity, allowing ideal sample size calculation. The post-sample size calculation generated an aggregated sample size of 79 with the associated 95% CI (71–88). Second, the evaluation method might have influenced the results, as a learning effect could have occurred, leading to improved performance in data analyses performed later. Nevertheless, it was shown that reliable results were obtained after a short training in MRI reading, regardless of the reader’s experience. This fact may allow the introduction of this imaging modality into daily clinical routine for general dentists who are less experienced in MRI imaging. Third, most study participants rarely had artifacts due to excluding all patients with implants in the molar region from this study. Thus, further investigations should consider refining the MRI sequence using zero TE or ultra-short TE methods, whereby these artifacts could be minimized.

Even though MRI currently cannot be applied in the daily clinical routine of private dental practice due to financial reasons, it is the only promising imaging modality that allows direct visualization of the NVB [47]. Initial efforts to enable out-of-hospital MRI examinations have been made possible by using newly developed bedside MRI scanners and specialized tools such as mandibular coils and radiofrequency (RF) coils [48,49], which represent an interesting option to make examinations more time and cost-efficient. Nevertheless, further improvements in terms of standardization are necessary for the future to enable the targeted use of MRI examinations in daily oral surgery procedures. Specifically, with the increasing debate on the need to optimize radiation dose according to the ALARA principle, especially in MTM surgery, these study’s findings demonstrate the importance and perspectives opening up due to the use of dental MRI. This evolving topic is about enabling diagnostically acceptable imaging performance with minimal radiation exposure while providing all necessary information for the upcoming surgical intervention.

From a clinical perspective, various imaging modalities will continue to be an essential part of dental diagnostic imaging. PAN will continue to be the method of choice for screening examinations as a primary diagnostic tool due to its practicability. In three-dimensional imaging, the selective use of dental MRI in indicated cases could usefully complement radiation-based CBCT imaging in the future. As the results show, dental MRI using “black bone” sequences has no significant limitations in diagnostic information, and by directly visualizing soft tissue, it even provides superior diagnostic information. A unique benefit could be the additional pre- and postoperative comparison of morphological changes of the nerve due to edema-related or iatrogenic trauma. However, routine use of dental MRI is currently not foreseeable for reasons of cost and effort. Although clinical protocols aim to minimize the risks associated with radiation exposure, further investigations are still needed to determine the unique limitations and indications for each imaging modality considering the “as low as reasonably achievable” and “as low as diagnostically acceptable” principles.

## 5. Conclusions

Dental MRI using black bone MRI sequences such as 3D-DESS MRI provides highly confidential, accurate, and reliable preoperative radiological assessment in MTM surgery, regardless of the investigator’s experience. Accordingly, the targeted use of Dental MRI and fused CBCT/MRI images in specific cases demonstrating a close positional proximity between the IAN/MC and the MTM may provide additional information allowing better prediction of surgical difficulties preoperatively.

## Figures and Tables

**Table 1:** The inter- and intra-rater agreement regarding the classification of the positional relationship between the IAN/MC and the MTM.

Positional Relationship	MRI Kappa Value (κ_W_ (95% CI))	CBCT Kappa Value (κ_W_ (95% CI))
Interrater Readout 1	0.482 (0.136–0.827)	0.558 (0.239–0.879)
Interrater Readout 2	0.654 (0.344–0.957)	0.574 (0.258–0.897)
Intrarater Reader A	0.514 (0.186–0.849)	0.589 (0.266–0.912)
Intrarater Reader B	0.534 (0.198–0.849)	0.565 (0.238–0.911)

**Table 2:** The interrater and intrarater agreement regarding the detectability of inflammatory processes is listed.

Inflammatory Process	MRI Kappa Value (κ_W_ (95% CI))
Interrater Readout 1	0.628 (0.349–0.905)
Interrater Readout 2	0.693 (0.413–0.958)
Intrarater Reader A	0.685 (0.416–0.945)
Intrarater Reader B	0.650 (0.367–0.945)

**Table 3:** The interrater and intrarater agreement regarding the fusion pattern analysis in the retromolar and molar region is expressed with the following kappa values.

Fusion Pattern	Fusion Pattern Retromolar (κ_W_ (95% CI))	Fusion Pattern Molar (κ_W_ (95% CI))
Interrater Readout 1	0.976 (0.935–1)	0.294 (−0.145–0.732)
Interrater Readout 2	0.973 (0.917–1)	0.759 (0.451–1)
Intrarater Reader A	0.871 (0.406–1)	0.736 (0.442–1)
Intrarater Reader B	0.785 (0.661–1)	0.216 (−0.207–0.639)

## Data Availability

The data presented in this study are available on request from the corresponding author. The data are not publicly available due to privacy restrictions.
